# Isolated Pneumopericardium: A Rare Complication Secondary to COVID-19 Infection

**DOI:** 10.7759/cureus.23431

**Published:** 2022-03-23

**Authors:** Sam Kara, Michael Talalaev, George D Yatzkan, Pamela Youssef, Kester Nedd

**Affiliations:** 1 Department of Neurology, Larkin Community Hospital Palm Springs Campus, Miami, USA; 2 Department of Pulmonary and Critical Care, Larkin Community Hospital Palm Springs Campus, Miami, USA; 3 Department of Pulmonary and Critical Care, Larkin University School of Medicine, Miami, USA

**Keywords:** covid-19 and heart, tension pneumopericardium, trauma, covid-19, pneumopericardium

## Abstract

Pneumopericardium in the setting of COVID-19 is a rare incident. Typically, COVID-19 manifests with respiratory failure, cytokine storm, and gastrointestinal and cardiac symptoms. Chest X-ray (CXR) shows patchy peripheral opacities in bilateral lung fields and computed tomography (CT) shows multifocal ground-glass opacities in a COVID-19 patient. However, CXR is relatively less specific when compared to CT. In this case report, we present a case of isolated pneumopericardium (without pneumomediastinum) in a young female patient with COVID-19 pneumonia. Not only is the mechanism of development of pneumopericardium in COVID-19 patients poorly understood, but it is also considered a bad prognostic factor that leads to mortality.

## Introduction

Pneumopericardium is a phenomenon resulting from air/gas in the pericardial cavity, and it is most frequently associated with pneumoperitoneum, pneumothorax, chest trauma, and pneumonia [1-3]. COVID-19 is a global epidemic that has claimed over 900,000 lives in the USA alone. While mortality has been devastating, complications of COVID-19 have been reported in many systems, such as neurological [4] and cardiogenic [5]. Spontaneous pneumopericardium and pneumomediastinum have been reported in COVID-19 patients [6-8].

Pneumopericardium is a rare condition associated with a high mortality rate and requires immediate diagnosis and treatment. It is mostly associated with a fistula between the pericardium and a gas-containing cavity, for example, the pleural space, trachea, or bronchial tree [9]. This rare condition may present as an asymptomatic incidental finding or nonspecific symptoms such as dyspnea or chest pain. Furthermore, this complication is self-limiting that requires, often, electrocardiogram and hemodynamic monitoring.

We encountered only one COVID-19 patient who developed spontaneous pneumopericardium without pneumomediastinum during their hospitalization at our facility. Pneumopericardium was defined by chest X-ray (CXR) only as the patient was medically unstable for a computed tomography (CT) scan. All radiological images were interpreted by a general radiologist and a cardiac radiologist. COVID-19 diagnosis was confirmed by a positive COVID-19 polymerase chain reaction (PCR) test, along with immunological testing of IgG and IgM [10].

## Case presentation

**Figure 1:**
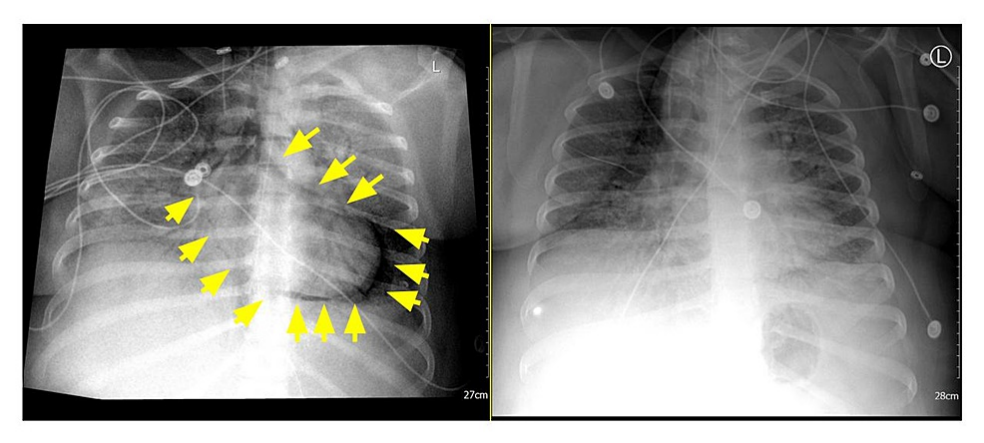
Chest X-ray 24 hours before and after pneumopericardium. Left: Chest X-ray shows ill-defined opacities and multifocal opacities. The enlarged cardiac silhouette is also present and may represent CHF pattern (yellow arrows: pneumopericardium). Right: X-ray shows linear lucency surrounding the cardiac shadow representing pneumopericardium.

A 35-year-old White female with a medical history of morbid obesity, obstructive sleep apnea (not on CPAP), asthma, and one week of COVID-19 infection presented to the ER at Larkin Community Hospital, Hialeah, Florida. The patient presented with one week of fever (103.5°F), chills, fatigue, body aches, progressively worsening shortness of breath, and nonproductive cough with left-sided pleuritic chest pain. She also had upper respiratory symptoms including cough, dyspnea, tachypnea, and shortness of breath at rest and is cyanotic with ambulation. The patient chest X-ray (CXR) showed bilateral diffuse ground-glass and consolidative opacification of bilateral lungs, she was hypoxemic (O2 saturation was 90%), and mechanical ventilation was initiated via nasal cannula 3 L/minute with O2 saturation improving to 98%. Her CXR and CT showed typical bilateral infiltrates consistent with COVID-19 pneumonia; laboratory findings showed elevated white cell count and lymphocytopenia and denied thoracic trauma. The patient was admitted to the intensive care unit (ICU), and initially, her respiratory status improved on 5 L nasal cannula oxygen and 10 mg daily oral methylprednisolone. On hospital day 4, the patient deteriorated with oxygen saturation of 89%, and invasive mechanical ventilation was initiated (assist-control (A/C) pressure control (PC); respiratory rate (RR), 20 breaths/minute; tidal volume (VT), 621 mL; inspiratory/expiratory ratio (I:E), 1.3:1; positive end-expiratory pressure (PEEP), 12; fraction of inspired oxygen (FiO2), 100%). She was placed on a nasogastric tube for feeding; blood pressure of 240/120 was controlled by hydralazine 40 mg, intravenous. On day 9 post-admission, the patient continued to have high oxygen demand and remained on mechanical ventilation (A/C PC; RR, 30 breaths/minute; PEEP, 22; FiO2, 100%; SpO2 at best, 87%). Chest X-ray showed that the patient had isolated pneumopericardium along with atypical viral pulmonary findings such as ground-glass opacities and consolidation [5]. Radiologic findings are presented in Figure 1.

## Discussion

Pneumopericardium is the accumulation of air in the pericardial space. Patients with pneumopericardium commonly present with chest pain, rapid heart rate, dyspnea, shortness of breath, pain extended from the chest to the back, or nausea [8]. The exact mechanism of pneumopericardium development is not well understood; however, it is speculated that the sequence of steps starts with the penetration of the extrapulmonary air into the thoracic cavity, followed by the rupture of the alveolar sac or alveolar duct, which allows thoracic air to enter into the interstitial tissue spaces where it could form primary interstitial emphysema [10]. Moreover, air could continue dissecting along the peribronchial connective tissue sheaths into the midline, where the pressure gradient drives further dissection along vascular sheaths toward the hilum, followed by the pericardial space between the parietal and visceral layers [8]. Pneumopericardium is commonly a consequence of trauma, positive pressure through mechanical ventilation, or invasive chest procedures, and it is unusual to observe as a result of viral or bacterial respiratory infections [8,10]. In respiratory infection such as COVID-19, the infection induces lung damage as a result of the inflammatory cascade, positive pressure injury, and hypercoagulability, which in turn increase the risk of developing pneumopericardium. Specifically, increased alveolar gradient pressure in the presence of diffuse alveolar injury increases the possibility of alveoli rupture, thus causing air dissection into the mediastinum, subcutaneous tissue, or cardiac cavity. Although most forms of pneumopericardium are associated with pneumomediastinum and are self-limiting, they can require invasive interventions and might be fatal [8-10]. Due to severe cardiorespiratory failure secondary to COVID-19, our patient died.

In severe cases of COVID-19, the lung goes under a hyperinflammatory process, and structural changes to the lung parenchyma increase intrathoracic pressure, which could act as inducing factors [11]. The deep cervical fascia, especially the visceral layers, are adjacent to the heart, and air from the lung could decompress and enter into the heart viscera. For such, elevated pressure in the deep cervical fascia is required; thus, it redirects air toward other planes. The rarity of these steps makes pneumopericardium a rare incident [11,12]. Nevertheless, elevated visceral pressure might cause spontaneous pneumopericardium, most commonly, in the presence of pneumomediastinum and extremely, on rare occasions, without it. Mechanically, pericardial tightness is weak at the venous sheaths and could present a gate for air entrance into it. Thus, we hypothesize that our patient's COVID-19 infection compromises tissue integrity leading to pneumopericardium. It is more common to encounter spontaneous pneumomediastinum; however, isolated pneumopericardium is extremely rare.

## Conclusions

Pneumopericardium is a rare complication and often requires management by the critical care teams. Although a rare complication by itself and, more often than not, is associated with pneumomediastinum, it is even rarer as a stand-alone phenomenon (without pneumomediastinum), let alone as a complication in COVID-19 patients. Pneumopericardium can bring about severe hypoxemia and devastating clinical consequences. The patient's clinical management was complicated by giant bulla formation, recent infection, and recurrent pneumothorax. These clinical issues can be efficiently diagnosed with simple imaging tools including chest X-ray, lung ultrasound, and chest CT. In the era of the COVID-19 pandemic, it is important to be vigilant to the unique pneumopericardium presentation to induce sufficient and timely treatment.
